# Human IP10-scFv and DC-induced CTL synergistically inhibit the growth of glioma in a xenograft model

**DOI:** 10.1007/s13277-014-1867-3

**Published:** 2014-05-10

**Authors:** Xuan Wang, Fang-Cheng Zhang, Hong-Yang Zhao, Xiao-Ling Lu, Yun Sun, Zhi-Yong Xiong, Xiao-Bing Jiang

**Affiliations:** 1 Department of Neurosurgery, Union Hospital, Tongji Medical College, Huazhong University of Science and Technology, 430022 Wuhan, China; 2 National Center for International Research of Biological Targeting Diagnosis and Therapy, Guangxi Key Laboratory of Biological Targeting Diagnosis and Therapy Research, Guangxi Medical University, Nanning, 530021 Guangxi China

**Keywords:** Human IP10-scFv, DC-induced CTL, Inhibit, Glioma

## Abstract

The epidermal growth factor receptor (EGFR) mutant of EGFRvIII is highly expressed in glioma cells, and the EGFRvIII-specific dendritic cell (DC)-induced tumor antigen-specific CD8^+^ cytotoxic T lymphocytes (CTLs) may hold promise in cancer immunotherapy. Interferon (IFN)-γ-inducible protein (IP)-10 (IP-10) is a potent inhibitor of angiogenesis and can recruit CXCR3^+^ T cells, including CD8^+^ T cells, which are important for the control of tumor growth. In this study, we assessed if the combination of IP10-EGFRvIIIscFv with DC-induced CTLs would improve the therapeutic antitumor efficacy. IP10-scFv was generated by linking the human IP-10 gene with the DNA fragment for anti-EGFRvIIIscFv with a (Gly_4_Ser)_3_ flexible linker, purified by affinity chromatography, and characterized for its anti-EGFRvIII immunoreactivity and chemotactic activity. DCs were isolated from human peripheral blood monocyte cells and pulsed with EGFRvIII-peptide, then co-cultured with autologous CD8^+^ T cells. BALB/c-nu mice were inoculated with human glioma U87-EGFRvIII cells in the brain and treated intracranially with IP10-scFv and/or intravenously with DC-induced CTLs for evaluating the therapeutic effect. Treatment with both IP10-scFv and EGFRvIII peptide-pulsed, DC-induced CTL synergistically inhibited the growth of glioma and prolonged the survival of tumor-bearing mice, which was accompanied by the inhibition of tumor angiogenesis and enhancement of cytotoxicity, thereby increasing the numbers of brain-infiltrating lymphocytes (BILs) and prolonging the residence time of CTLs in the tumor.

## Introduction

Malignant gliomas are one of the most devastating tumors encountered in current clinical practice. Despite improved surgical management and a multimodal treatment of concomitant radiotherapy and chemotherapy followed by adjuvant chemotherapy with temozolomide (TMZ), the prognosis remains poor, with median overall survival of less than 15 months [1]. Therefore, studies actively focus on testing new therapeutic approaches, including immunotherapy. The recognition that lymphocyte infiltration into primary brain tumors is a favorable prognostic factor for survival has inspired a variety of approaches to utilize the immune system for the treatment of these tumors [2].

Adoptive immunotherapy using antigen-specific CD8^+^ cytotoxic T lymphocytes (CTL) provides a promising approach for the treatment of cancers and infectious diseases [3]. One major obstacle to its broad application has been the lack of reproducible and cost-effective methods for generating clinically relevant numbers of antigen-specific CTLs [4]. The generation of antitumor-specific T cells ex vivo for adoptive T cell therapy requires the selection of an appropriate tumor rejection antigen, a means to present that antigen and conditions that support the generation of antitumor-specific T cells that are both long-lived and functional. Based on the large numbers of potential tumor rejection antigens that have been characterized, the choice of the antigen is not likely to be a major obstacle [5, 6]. In contrast, the choice of an optimal antigen-presenting cell (APC) seems to be much more challenging.

However, the choice of antigen also appears to be much more challenging. EGFRvIII is the most prevalent of several epidermal growth factor receptor (EGFR) mutations found in human glioma and is expressed in 20–25 % of glioblastoma (GBM) cases [7]. While EGFRvIII drives enhanced tumorigenicity through constitutive and unattenuated activation [8], its expression has not been detected in any normal tissue examined to date [9], thus making this aberrant receptor a suitable target for glioma immunotherapy.

Dendritic cells are the most potent professional APCs that exist in the immune system. Once activated, they process antigen material and present it on the surface to other immune cells in the system [10, 11]. For example, dendritic cells (DCs) can present antigen to initial T lymphocytes and in turn activate and induce T lymphocytes into antigen-specific CTLs that target tumor cells [12]. Among the activated CTLs, CD8^+^ CTLs are believed to be the major effector cells that kill target cells [13, 14]. Furthermore, matured DCs can secrete interleukin-12 (IL-12) and reduce production of interleukin-10 (IL-10), which inhibits the CD8^+^ T cell immunologic response [15, 16].

Currently, a number of immunotherapeutic approaches targeting the unique EGFRvIII antigen are under investigation. Given the technical difficulty and relatively high cost of DC-based vaccination therapy, the most promising and practical active vaccination format to date is a peptide derived from the novel fusion junction amino acid sequence. Heimberger et al. [17] demonstrated that immunization of mice with DCs mixed with a 14-amino-acid peptide, representing an EGFRvIII fusion sequence, resulted in prolonged survival of mice with EGFRvIII expressing tumors as well as long-lasting humoral immunity.

Chemokines are small secreted proteins that activate G protein-coupled receptors, resulting in the activation of several distinct signaling cascades and leading, most importantly, to directed migration of cells along the chemokine gradient [18]. Interferon (IFN)-γ-inducible protein (IP)-10 is a CXC chemokine that is secreted by the endothelium, epithelium, fibroblasts, keratinocytes, and monocytes [19, 20]. IP-10 was initially characterized as a chemoattractant for CXC receptor (R) 3-expressing T lymphocytes, and it also exerts chemotactic activity for monocytes. However, with the exception of chemotaxis, IP10 is a potent inhibitor of angiogenesis and displays some thymus-dependent antitumor effects [21, 22]. Therapy with CXCL10 is effective in reducing the rate of tumor growth, whereas it fails to induce tumor complete regression, which suggests that further treatment may require supplemental combination therapies that directly target tumor cells.

Antibody-mediated targeting of drugs and genes to specific cells population has been a long-term research interest in the field and there has been some success in the targeting of tumors with antibodies or engineered derivatives [23, 24]. However, the fast clearance of small antibody fragments and poor penetration of antibodies into the tumor requires multiple injections and limits their therapeutic potential. The use of engineered therapeutic single-chain Fv (scFv) fragments that target tumor cells might overcome this problem. This scFv was previously well characterized [25, 26] and shown to specifically target EGFRvIII-expressing U87 glioma cells in vivo [27].

In this study, we describe the construction of a novel antibody–chemokine fusion protein, called IP10-EGFRvIIIscFv, for targeting tumor cells and triggering a specific immune response against tumors by linking the chemokine IP-10 to a scFv fragment specific for EGFRvIII combined with EGFRvIII-specific DCs. It was hypothesized that IP10-EGFRvIIIscFv would be an effective vehicle for targeted delivery of cytokines to the sites of the tumor and at the same time may improve the concentration of DC-induced CTL that infiltrates into the tumor.

## Materials and methods

### Cell lines, mice, and healthy volunteers

The U87 human glioblastoma cell line, the U87 line stably expressing EGFRvIII (U87-MGvIII), human embryonic kidney 293 (HEK-293) cell line, mouse NIH 3 T3 embryonic fibroblast cell line, and human Hep3B hepatocellular carcinoma cell line were obtained from our laboratory. All cell lines were cultured in Dulbecco’s modified Eagle’s medium (DMEM; GIBCO, Invitrogen) supplemented with 2 mM l-glutamine, 100 U/ml penicillin, and 10 % fetal bovine serum (FBS; Invitrogen). Six-week-old female BALB/c-nu mice (SPF) were obtained from the pathogen-free animal facility of the Tongji Medical College, Hua Zhong University of Science and Technology. All mouse procedures and isolation of peripheral blood mononuclear cells (PBMCs) from five healthy HLA-A0201 donors were performed in accordance with institutional protocol guidelines at the Tongji Medical College. The experimental protocol was established according to the guidelines of NIH Animal Research and Care and approved by the Ethics Committee of the Union Hospital of Tongji Medical College.

### Cloning of expression plasmids

The nucleotide sequence encoding human IP-10 complementary DNA (cDNA) (Gene Bank Accession No. 02530) was amplified using the following parameters: 22 cycles of denaturation at 95 °C for 30 s, primer annealing at 56 °C for 30 s, and primer extension at 72 °C for 1 min. The following pair of primers was used: forward, 5′-TCCGCTCGAGGCCACCATGCATCATCATCATCATCATATGAATCAAACTGCCATTCTG-3′and reverse, 5′-ATCGGAATTCTCATTTGATTTCCAGCTTGGTGC-3′. The PCR products were ligated into the T overhang-cloning site of a TA cloning vector (Invitogen). The cDNA (Gene Bank Accession No. U76382.1) encoding EGFRvIIIscFv (MR1) was previously obtained using a phage display library [25]. We then introduced the sequences encoding a flexible linker of (Gly_4_Ser)_3_ and helix-histidine tag (His_6_-tag), respectively, and sub-cloned IP10- EGFRvIIIscFv into an XhoI- and EcoRI-digested GV219 vector (Gene Chem).

### Transfections and generation of stable cell lines

We used the conventional lipofectamine method to transfect NIH3T3 cells. Prior to transfection, NIH3T3 cell lines were grown at 37 °C and 5 % CO_2_ in DMEM containing 10 % FBS. When they reached approximately 70–80 % confluency, vectors were transfected into all systems using the Lipofectamine TMX reagent (Invitrogen). Briefly, Lipofectamine TMX was mixed with plasmid DNA (0.68 μg/μl) in Opti-MEM medium (Invitrogen) according to the manufacturer^’^s recommendations. After culturing the cells for 48 h at 37 °C, the transfected cells were added to 24-well plates (Costar, USA) at a density of 1 × 10^5^ cells/well and selected in 400 μg/ml G418 (Invitrogen). Stable cell lines were obtained in 2–4 weeks post-transfection. A combination of fluorescent activated cell sorting (FACS) (FACS Aria, BD Biosciences) and single colony selection by limiting dilution were used to select for the highest expressing cells.

### Expression and purification of the fusion protein

The chosen highest expressing NIH3T3 cells were grown to 80–90 % and induced with 1–2 μg/ml doxycycline and 10 mM sodium butyrate (Sigma) for 24–36 h. In order to evaluate the cell surface or intracellular expression of the fusion protein, cells were harvested by incubating them in phosphate-buffered saline (PBS) containing 0.5 m MEDTA and washed with PBS containing 0.1 % bovine serum albumin (BSA). For purification, cells with stable expression of the fusion protein were collected by centrifugation at 300*g* for 10 min at 4 °C in an Eppendorf high-speed centrifuge. The cell pellet was then washed three times with PBS and resuspended in buffer containing 50 mM HEPES (pH 7.4), 1 mM EDTA, 1 μM pepstatin, 100 μM leupeptin, and phosphatase inhibitor cocktail (1:100, Sigma). The cells were sonicated five times for 10 with 15 s intervals, and the cell lysate was further centrifuged at 12,000*g* for 20 min at 4 °C. Supernatant containing protein was then precipitated with 50 % ammonium sulfate and applied to a Ni-chelating His Trap column (Amersham-Pharmacia Biotech) equilibrated with PBS (pH 7.4) containing 20 mM imidazole according to the manufacturers protocol. The bound protein was eluted with 5 ml of 0.1 M imidazole in PBS followed by dialysis against PBS. The protein concentration was determined by the BCA kit (Pierce) according to the manufacturer’s protocol using BSA as a standard. Fractions collected from the chromatography steps were analyzed on a 12 % sodium dodecyl sulfate (SDS) polyacrylamide gel and stained with silver nitrate.

### ELISA assay for affinity binding test

The affinity binding of the IP10-scFv purified fusion protein was measured by ELISA. A 13-amino-acid peptide with a terminal cysteine (LEEKKGNYVVTDHC) [28] consisting of an epitope recognized by the anti-EGFRvIIIscFv antibody was synthesized and cross-linked with OVA as an antigen at a concentration of 0.5 μg/ml (0.05 μg/well) for coating ELISA plates. Wells were coated with BSA at a concentration of 0.5 μg/ml under the same conditions as a negative control. After overnight incubation at room temperature, the plate was washed three times with 100 μl of PBS-tween (PBST) and blocked with a 1 % BSA solution in PBS containing 0.05 % Tween 20 and finally washed with PBST. Various concentrations (0 to 2 μg/ml) of IP10-scFv were added to individual wells in triplicate, and the plates were incubated for at least 1 h at room temperature. After washing, the remaining IP10-scFv was detected by a biotinylated anti-6 × Histagmonoclonal antibody (mAb) and visualized using horseradish peroxidase (HRP)-conjugated avidin (Peprotech) and substrate of ABTS (Sigma) by measuring the absorbance at 405 and 650 nm as a correction wavelength. For analysis, A405nm values after correction were plotted against the IP10-scFv fusion protein concentration using Graphpad Prism software. Data were fitted by nonlinear regression to a hyperbolic function [29] (*A* = (*A*_max_ *c* / (*K*_*D*_ + *c*)), where *A* is the measured signal, *A*_max_ is the signal for saturated binding, and *c* is the protein concentration. The apparent *K*_*D*_ values were determined from this equation.

### Antigen binding assay

U87 cells stably expressing EGFRvIII were washed with PBS containing 1 % BSA (pH 7.4) and incubated with 100 ng IP10-scFv fusion protein for 1 h at 4 °C followed by 1 μg/ml anti-His_6_mAb. The cells were washed and stained with fluorescein isothiocyanate (FITC)-conjugated rabbit antimouse IgG (Abcam) and analyzed by immunofluorescence under a fluorescent microscope or by flow cytometry. An isotype mAb to lipopolysaccharide (LPS) and the EGFRvIII negative U87wt cells were used as the negative controls, respectively.

### In vitro preparation of EGFRvIII peptide CTLs

PBMCs from five healthy HLA-A0201 donors were separated using Lymphoprep™ Human Lymphocyte density gradient medium (Axis-Shield). Briefly, the separated mononuclear cells were cultured in RPMI-1640 medium supplemented with recombinant granulocyte-macrophage colony stimulating factor (GM-CSF, 1,000 IU/ml, Peprotech, USA) and recombinant interleukin-4 (IL-4, 500 IU/ml; Peprotech, USA) for 7 days with fresh cytokine medium replaced every 2–3 days. On day 5, the immature DCs were activated by supplementation of tumor necrosis factor α (TNF-α, 1,000 IU/ml; PeproTech) in the culture medium. At the end of cell culture (day 7), the mature DCs were harvested for subsequent experiments. During the cultivation, DCs were observed by phase-contrast microscopy and analyzed for surface molecular expression by flow cytometry (date were not shown).

EGFRvIII peptide-specific CTLs were generated in vitro according to the method described by Wu et al. [30]. Briefly, mature DCs were pulsed with EGFRvIII peptides in X-VIVO15 media for 4 h at 37 °C, then washed twice in HBSS and irradiated 3,500 rad in a cesium irradiator, and cultured with autologous purified CD8+ T cells at 1:20 ratio in 48 well plates. The T cells were individually re-stimulated with autologous DCs pulsed with the priming peptide every 9 days. Starting on day 12, the T cell cultures were fed with fresh X-VIVO15 medium containing 50 U/ml of rh-IL-2 (PeproTech) every 3 days. The Elispot assay and CTL assay were performed 7 days after three rounds of in vitro stimulation with peptide-pulsed DCs.

### Cellular migration assays in vitro

Migration assays using the IP10-scFv fusion protein were performed in a 24-well Transwell chamber (5-μm pore size; Corning Inc.) coated with 10 mg/ml of fibronectin (Sigma-Aldrich) on the bottom of the upper chamber. The DC-induced CTLs were suspended in RPMI 1640 containing 1 % BSA and applied at a density of 1 × 10^5^/well. Then, 200 μl of the cell suspension was placed into the upper Transwell chamber and 100 μl (1 ng/μl) aliquots of the refolded IP10-scFv, 100 ng recombinant human IP-10 (Peprotech), 100 ng anti-EGFRvIII monoclonal antibody (Zymed), and PBS serially diluted in chemotaxis medium (RPMI1640 with 1 % BSA) were placed in the lower chamber. The chambers were then incubated for 3 h at 37 °C in a humid atmosphere of 5 % CO_2_. After incubation, the number of cells that migrated to the lower chamber and mixed uniformly was determined with crystal violet staining followed by counting under a light microscope (16 selected high-power fields). The results are expressed as the chemotaxis index, which was calculated using the following formula: chemotaxis index = migration in response to chemokine / migration to control medium. The index represents the mean of the migration performed in triplicate.

### Tumor challenge

For intracranial (i.c.) tumors, a suspension of 1 × 10^7^/ml U87-EGFRvIII cells in 5 μl PBS were stereotactically injected through an entry site at the bregma located 2 mm to the right of the sagittal suture and 3 mm below the surface of the skull of anesthetized mice (6-week-old female mice BABL/C-un, SPF) using a Reword stereotactic frame (Reword instrument). We then divided the mice into four groups (*n* = 10 each). One group of mice received IP10-scFv fusion protein (i.c.) and EGFRvIII peptide-pulsed, DC-induced CTLs (i.v.) on day 7, 14, and 21 after tumor establishment. Mice in the second group were only injected with IP10-scFv fusion protein (i.c.) on the same day time of tumor challenge. The third group received EGFRvIII peptide-pulsed, DC-induced CTLs (i.v.) on the same day. Mice in the fourth group were injected with PBS (i.c.) as a negative control on the same day. However, mice were monitored carefully for changes in psychosis and pathologic signs associated with cerebral problems, such as hemiparesis, loss of appetite, or altered grooming habits. Mice were euthanized by CO_2_ to obtain tumor tissues and determine tumor volumes using Vernier calipers at selected time points according to the formula: d1 × (d2)^2^ × 0.5 (d1 = largest diameter, d2 = perpendicular diameter). Kaplan–Meier survival curves were prepared and median survival times were determined for all groups. Survival differences were assessed using the log-rank Mantel–Cox method.

### Cytotoxic assay

The cytotoxic activity of the DC-induced CTLs was determined using the ^51^Cr-release assay with minor modifications. Briefly, the brain-infiltrating lymphocytes (BIL) were isolated from mice receiving IP10-scFv and DC-induced CTLs, IP10-scFv, DC-induced CTLs, or PBS on day 14 post-inoculation as previously described [31]. The BIL (1 × 10^5^/well) were stimulated with U87-EGFRvIII cells in 10 % FBS RPMI1640 for 5 days, and the activated cells were used as effector cells. In addition, U87-EGFRvIII target cells as well as HEK-293, Hep3B, and U87-wt cells (1 × 10^6^) were labeled with 40μCi^51^Cr-sodium chromate (Amersham Pharmacia Biotech) at 37 °C for 2 h. After washing with PBS, the target cells were incubated in triplicate with activated effectors at different ratios of 40:1, 20:1, 10:1, and 5:1 in 96-well U-bottom microtiter plates (Nunc, Roskilde, Denmark) for 4 h, respectively. The target or effector cells alone were used as the controls. The target cells were treated with 1 % Triton X-100 and used as the maximum release of ^51^Cr. The plates were centrifuged at 600×*g*, and the supernatant was counted using a gamma counter. The percentage of glioma-specific cytotoxicity was calculated using the following formula: [[CPM (experimental) − CPM (spontaneous)] / [CPM (maximum) − CPM (spontaneous)]] × 100.

### Quantification of tumor vessels and CD8^+^ T cells

The U87-EGFRvIII tumor tissue sections were permeabilized using 0.5 % Triton X-100 for 10 min and blocked with 3 % BSA for 20 min. The rabbit polyclonal antihuman CD31 antibody (ab28364, Abcam) and mouse monoclonal antihuman CD8 antibody (ab17147, Abcam) was used at 1:50 and1:200 dilutions, respectively, in PBS at 4 °C overnight. The following day, sections were washed three times with PBS and incubated subsequently in goat antirabbit Alexa Fluor 594 red fluorescent (dilution 1:1,000, BD), rabbit antimouse FITC (dilution 1:1,000, BD) for 1 h at room temperature. The sections were then washed three times with PBS and the number of cells per high-powered field in several sections from multiple animals was determined. From these data the mean and standard error of the mean (SEM) were calculated. The data were subjected to statistical analysis (one-tailed student’s *T*-test).

### Statistical analysis

Data are expressed as mean ± standard deviation (SD). The difference among different groups was analyzed by one-way analysis of variance (ANOVA) and post hoc Bonferroni correction, and the difference between two groups was analyzed by Student’s *t* test using SPSS version 16.0 software (SPSS, USA). The survival of individual groups of mice was analyzed by the Kaplan–Meier and log-rank Mantel-Cox methods. A *P* value of <0.05 was considered statistically significant.

## Results

### Construction of the IP10-scFv expression vector

**Fig. 1:**
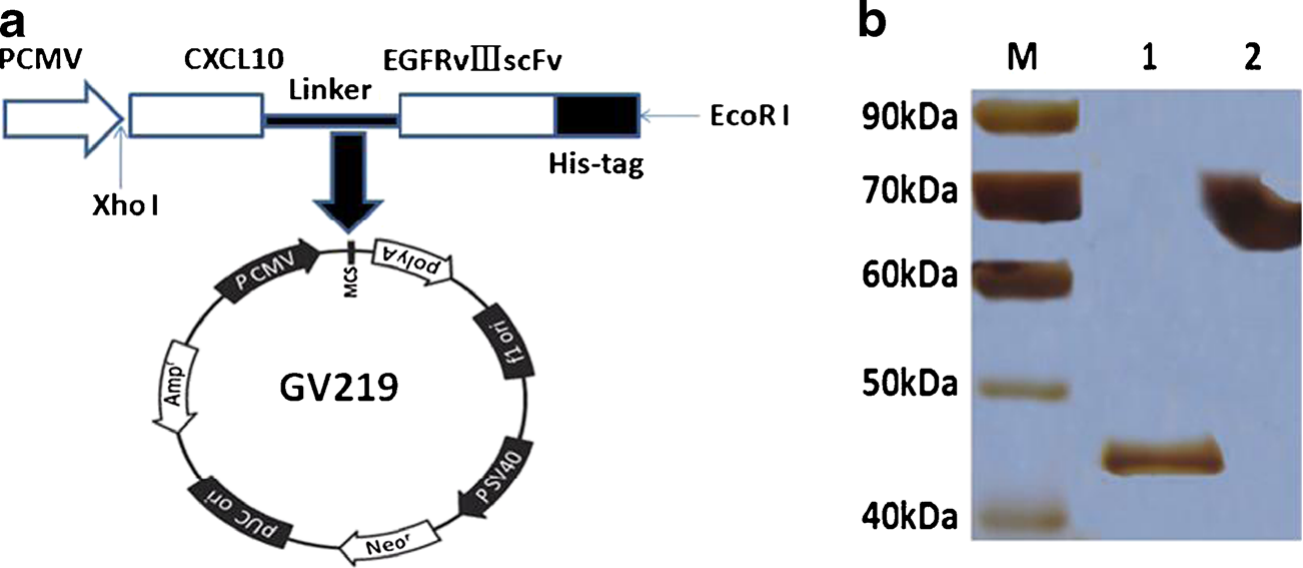
Generation and characterization of recombinant IP10-scFv**. a**The IP10-scFv gene was inserted into GV219 under the control of the PCMV promoter as a XholI/EcoRI fragment and amplified from the plasmid GV219IP10-scFv. **b** The expressed IP10-scFv was expected to be a fusion protein of 371 amino acids with a molecular weight of 44 kDa, which contains a His-tag at the C-terminus for detection and purification. The purified protein was then subjected to 12 % SDS-PAGE and stained with Ag. *Lane M*, mid-range protein molecular weight markers (kDa). *Lane 1*, the purified IP10-scFv fusion protein. *Lane 2*, BSA

The recombinant expression vector GV219IP10-scFv was constructed for the production of IP10-scFv fusion protein, in which the human IP10 gene was linked to the N-terminal of the anti-EGFRvIII-scFv via a (Gly_4_Ser)_3_ flexible linker, which facilitated both correct folding of the scFv and IP10 proteins [32, 33]. In addition, a His-tag was introduced at the C-terminal of IP10-scFv for detection and affinity purification (Fig 1a). The resultant recombinant protein was expected to be a fusion protein containing 371 amino acids.

### Expression and purification of IP10-scFv fusion protein

The recombinant plasmid GV219IP10-scFv was transfected into NIH3T3 cells and stable transfectants were selected and expanded. The recombinant proteins was then purified from ascites fluids using a Ni-chelating affinity column and verified by SDS-PAGE and Ag staining. Expression of the IP10-scFv fusion protein was confirmed in the transfected NIH3T3 cells, and the apparent molecular weight of the recombinant protein on SDS-PAGE gel was approximately 44 kDa (Fig. 1b), which was equivalent to the theoretical value of 47 kDa.

### Binding of recombinant IP10-scFv to EGFRvIII

The binding properties of the recombinant IP10-scFv were analyzed by ELISA. The EGFRvIII was coated onto an ELISA microplate, and the IP10-scFv protein bound to it was detected with a monoclonal antibody against 6 × His-tag. IP10-scFv was capable of binding to EGFRvIII in a dose-dependent manner with saturation at IP10-scFv protein concentrations greater than 450 nM. The apparent dissociation constant (*K*_*D*_) was found to be 1.02 ± 0.06 × 10^−8^ M. However, strong signals were obtained at the lowest IP10-scFv concentrations tested in the assay, which complicated quantitative evaluation of the data.

### Antigen binding of IP10-scFv fusion protein

**Fig. 2:**
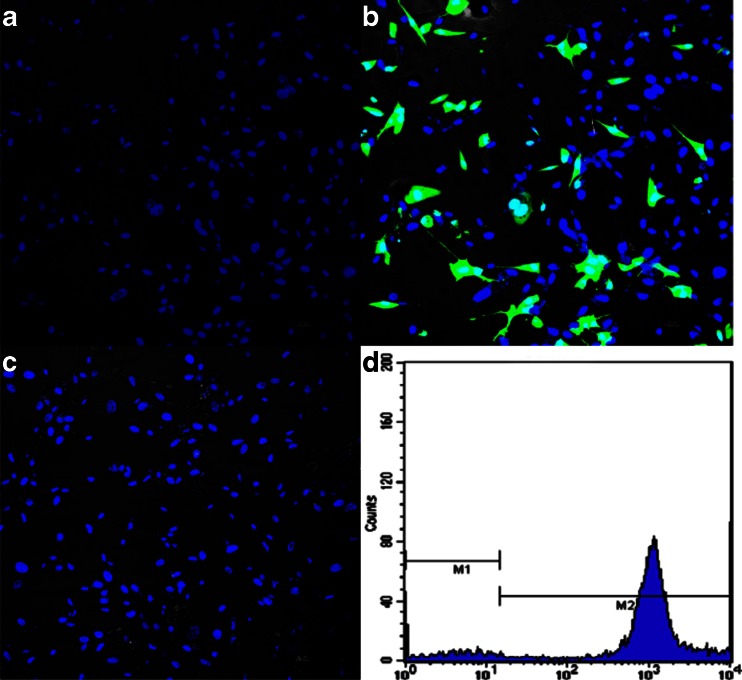
Antigen binding of the refolded IP10-scFv to EGFRvIII. U87-EGFRvIII cells were incubated separately with IP10-scFv (**a**) or IgG (**b**) followed by anti-His_6_ mAb incubation. U87wt cells were incubated with IP10-scFv as a control group (**c**). The percentage of refolded IP10-scFv bound to U87-EGFRvIII by flow cytometry analysis (**d**) was71.3 %

To determine the ability of recombinant IP10-scFv binding to EGFRvIII, the binding of IP10-scFv, IgG to U87-EGFRvIII cells, and U87wt cells were analyzed by immunofluorescence under a fluorescent microscope or by flow cytometry analysis. The results showed that only IP10-scFv bound specifically to the surface of U87- EGFRvIII cells, while no binding was detected from either the IgG control or IP10-scFv incubated withU87wt cells (Fig. 2).

### CTL migration in response to IP10-scFv

**Fig. 3:**
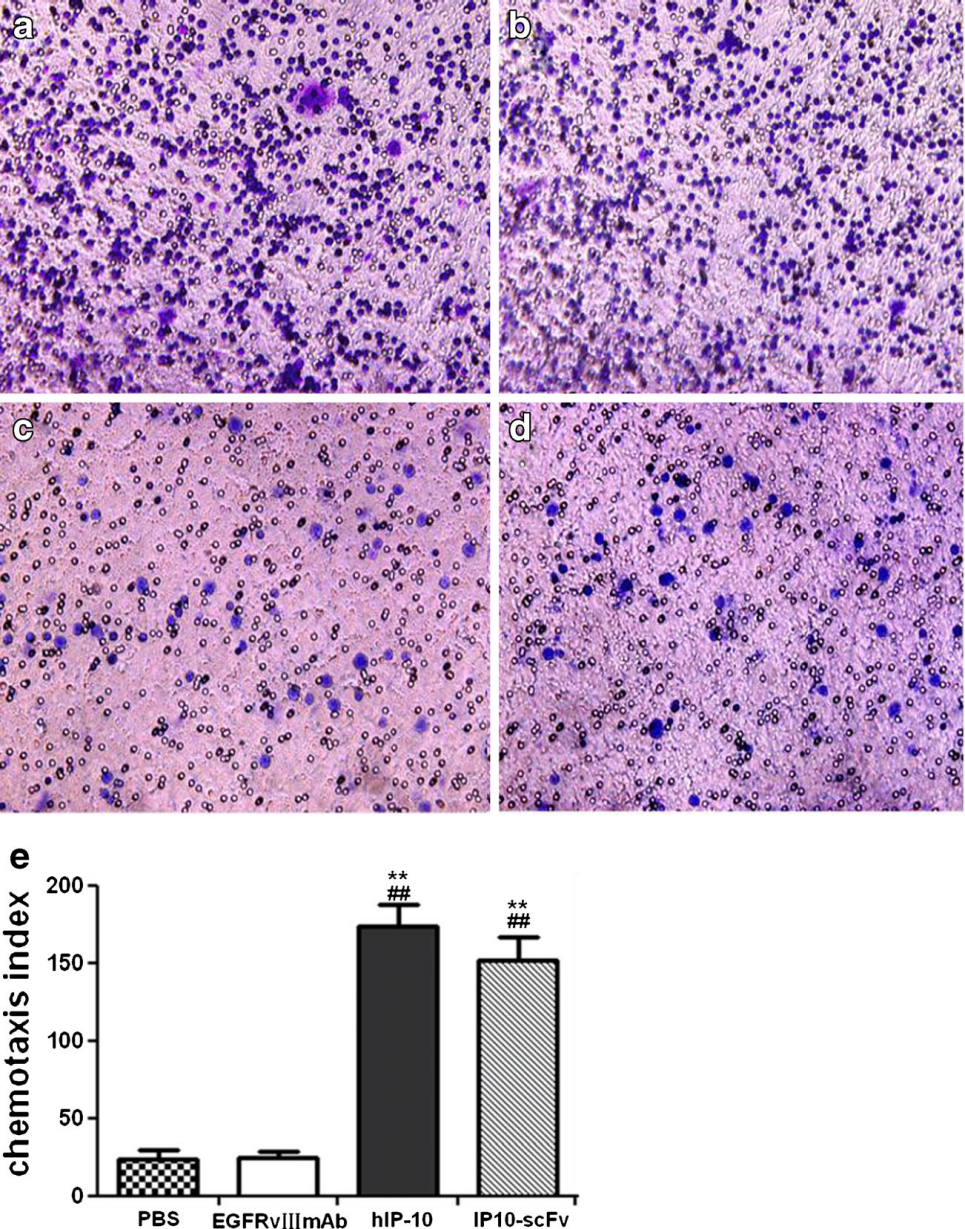
The chemotactic activity of the purified IP10-scFv to EGFRvIII peptide-pulsed, DC-induced CTLs. **a** 100 ng refolded fusion protein IP10-scFv, **b** 100 ng recombinant human IP10, **c** 100 ng anti-EGFRvIII monoclonal antibody, or **d** PBS. **e** Data were expressed as the chemotaxis index of two groups of CTL cells from three separate experiments. There was no difference between IP10-scFv and hIP10 (*P* > 0.05) or in the PBS and anti-EGFRvIII mAb groups (*P* > 0.05). However, the chemotaxis index was significantly higher in the IP10-scFv or hIP-10 groups than the PBS or anti-EGFRvIII mAb groups (*P* < 0.001)

To determine whether the IP10-scFv fusion protein could facilitate the transendothelial chemotaxis required for recruitment of CTL to the target cells, we prepared DC-induced CTLs in vitro. The chemotactic activity of IP10-scFv was then tested for the migration of CTLs in the transwell migration assays. As shown in Fig 3, the chemotactic activities of IP10-scFv were similar to that of recombinant human IP10 (*P* > 0.05). Importantly, there was no detectable chemotactic activity for the anti-EGFRvIII mAb or PBS controls. Collectively, these data clearly indicate that the purified recombinant IP10-scFv protein retains immunoreactivity of the scFv against the EGFRvIII mAb and the chemotactic activity of IP10.

### Antitumor efficacy of the combination of EGFRvIII peptide-pulsed, DC-induced CTLs and IP10-scFv

**Fig. 4:**
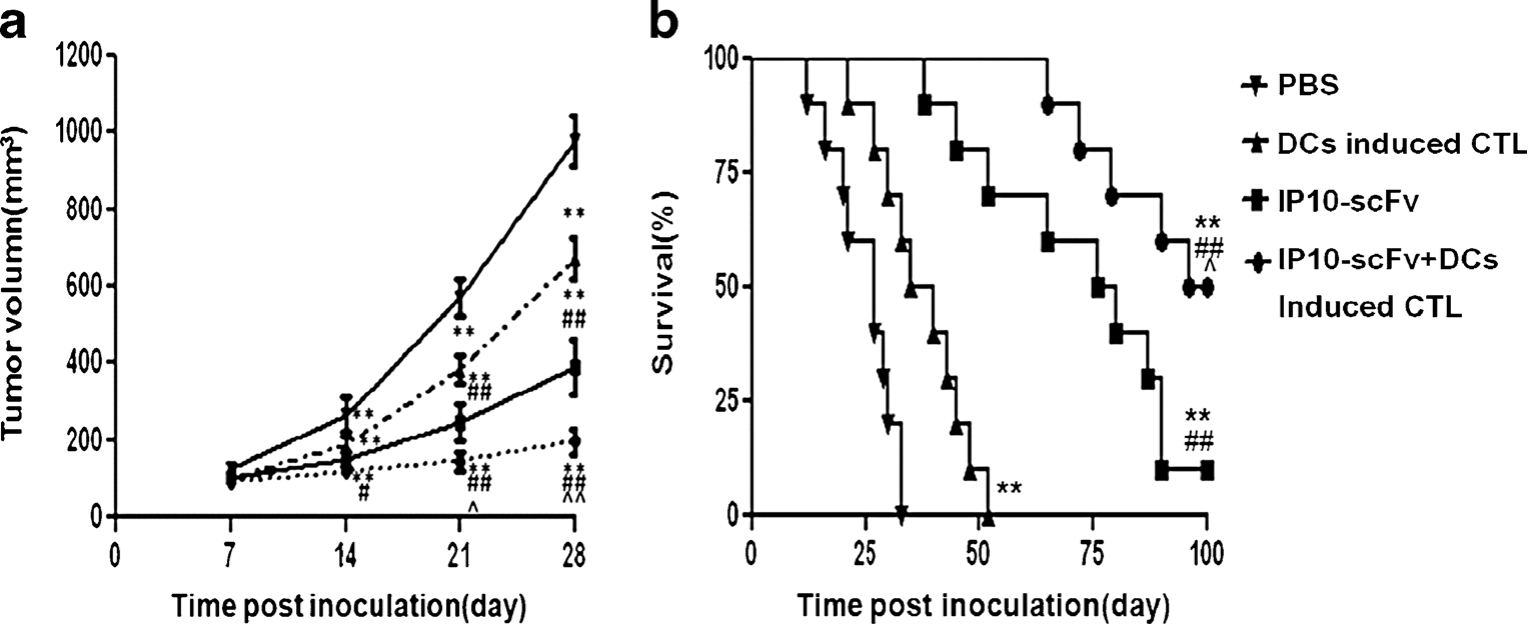
Inhibition of tumor growth and survival analysis. **a**. The kinetics of tumor growth. Data are expressed as mean ± standard deviation (SD) of the tumor volumes of each group of mice (*n* = 10 per group per time point). **b**. The survival curves of tumor-bearing mice were estimated using the Kaplan–Meier method. Data are expressed as the mean percentage of each group of mice (*n* = 10 per group) that survived throughout the period. *P < 0.05, **P < 0.01 vs. the PBS group; ^#^ *P* < 0.05, ^##^ *P* < 0.01 vs. the EGFRvIII peptide-pulsed, DC-induced CTL group; ^^^ *P* < 0.05, ^^^^ *P* < 0.01 vs. the IP10-scFv group

We next examined the efficacy of the IP10-scFv together with EGFRvIII peptide-pulsed, DC-induced CTLs in a murine intracerebral glioma model. In the present study, BABL/c nu mice received adoptive immunotherapy by injecting EGFRvIII peptide-pulsed, DC-induced CTLs (i.v.) and IP10-scFv (i.c.) beginning 7 days after tumor implantation. Control groups were only injected with IP10-scFv (i.c.), CTLs (i.v.), or PBS (i.c.), respectively. Vaccinations were repeated every 7 days for a total of three doses. Kaplan–Meier survival plots were drawn, and median survival times were determined for all groups. We found that the tumor volumes of each group expanded at a near-linear rate as determined by measuring their size every 7 days (Fig. 4a). In contrast, the rate of tumor growth in animals treated with DC-induced CTLs alone or IP10-scFv fusion protein alone was significantly decreased compared to the PBS control-treated animals (*P* < 0.01, both comparisons). In addition, the combination treatment of DC-induced CTLs plus IP10-scFv reduced the tumor growth more effectively than DC-induced CTLs or IP10-scFv alone (*P* < 0.01, both comparisons). Similarly, we monitored the overall survival of tumor-bearing mice and found that while all of the mice treated with PBS or DC-induced CTLs alone died between day 33 and 52 post-inoculation, less than 50 % of the mice that had been treated with IP10-scFv or with both IP10-scFv and DC-induced CTLs had died by day 65 post-inoculation (Fig. 4b). Moreover, we found that 10 and 50 % of mice survived for the entire observation period (up to 100 days post-inoculation) in the groups treated with IP10-scFv and IP10-scFv/DC-induced CTLs, respectively. The effect of treatment with IP10-scFv and DC-induced CTLs on prolonging the survival of tumor-bearing mice was significantly greater than that of treatment with DC-induced CTLs alone (*P* < 0.001) or with IP10-scFv alone (*P* < 0.05). Therefore, treatment with both IP10-scFv and DC-induced CTLs synergistically inhibited the growth of implanted glioma in vivo and prolonged the survival of tumor-bearing mice.

### Cytotoxicity of CTLs in mice

**Fig. 5:**
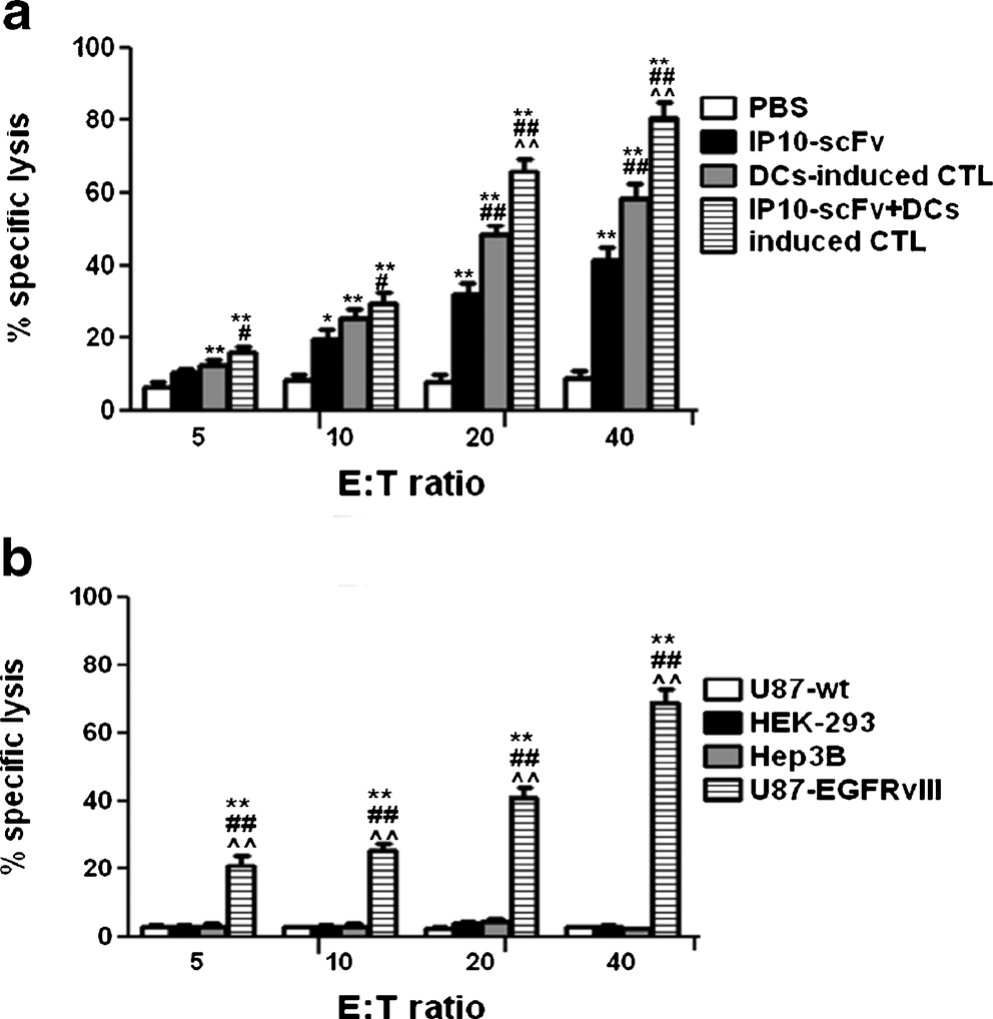
The glioma-specific cytotoxicity. **a** The BIL were isolated from the IP10-scFv/EGFRvIII peptide-pulsed, DC-induced CTLs, IP10-scFv, EGFRvIII peptide-pulsed, DC-induced CTLs, or PBS-treated mice on day 14 post-inoculation. **b** The BIL (1 × 10^5^/well) were stimulated with U87wt, HEK293, Hep3B, and U87-EGFRvIII cells in vitro for 5 days. Data are expressed as mean ± SD of the percentages of glioma-specific cytotoxicity in different groups of mice from three separate experiments. *P < 0.05, **P < 0.01 vs. the PBS and U87wt groups; ^#^ *P* < 0.05, ^##^ *P* < 0.01 vs. the IP10-scFv and HEK293 groups; ^^^ *P* < 0.05, ^^^^ *P* < 0.01 vs. the EGFRvIII peptide-pulsed, DC-induced CTLs and Hep3B groups

We next demonstrated tumor-specific CTLs against U87-EGFRvIII cells with a standard 4h^51^Cr release assay using BIL from the four treated groups, As shown in Fig 5a, there was a low level of cytotoxicity in the mice that had been treated with IP10-scFv alone, which was similar to that in the control mice, regardless of the ratios of effectors to targets. In contrast, the percentages of U87-EGFRvIII lysed by the BILs from mice treated with EGFRvIII peptide-pulsed, DC-induced CTLs increased significantly as the ratio of effectors to targets increased (*P* < 0.05–0.001), and the frequency of U87-EGFRvIII cells lysed by the BILs from mice treated with IP10-scFv/CTLs was significantly higher (*P* < 0.01). Similarly, the in vitro generated CTLs had potent cytotoxicity against glioma cells, but failed to kill other tumor cells and non-tumor cells (Fig. 5b).

### Effect of IP10-scFv + EGFRvIII peptide-pulsed, DC-induced CTLs on tumor vessel and CD8^+^ T cell counts

**Fig. 6:**
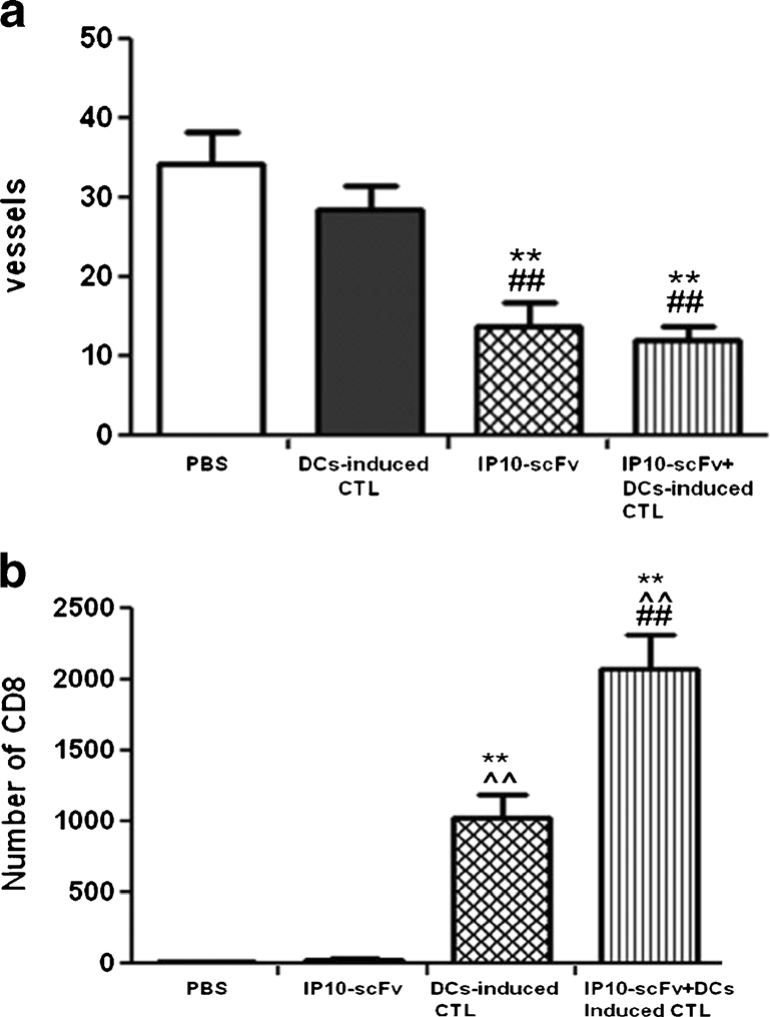
Immunofluorescent staining of CD31 and CD8 in tumor tissue of mice. **a** Quantification of CD31 expression in each group. In the IP10-scFv + EGFRvIII peptide-pulsed DC-induced CTL group, the number of vessels was the lowest and was not different than theIP10-scFv group (*P* > 0.05). However, the number of CD31-positive vessels was the highest in the PBS group. **b** Quantification of CD8 expression in each group. The IP10-scFv/EGFRvIII peptide-pulsed, DC-induced CTLs can attract the most CD8+ T cells to infiltrate the tumor. In contrast, there were no obvious differences in the number of CD8+ T cells between the PBS and IP10-scFv-treated groups. Data are expressed as mean ± SD of the percentages of CD31 and CD8 in different groups of mice from three separate experiments. *P < 0.05, **P < 0.01 vs. the PBS group; ^#^ *P* < 0.05, ^##^ *P* < 0.01 vs. the EGFRvIII peptide-pulsed DC-induced CTL group; ^^^ *P* < 0.05, ^^^^ *P* < 0.01 vs. the IP10-scFv group

Immunofluorescence studies were performed on intracerebral U87-EGFRvIII tumors from mice vaccinated with four treatments (IP10-scFv/EGFRvIII peptide-pulsed, DC-induced CTLs, IP10-scFv, EGFRvIII peptide-pulsed, DC-induced CTLs, or PBS). As shown in Fig 6a, CD31 staining was used to detect vessels in U87-EGFRvIII tumors and showed a significant decrease in the number of tumor vessels in mice treated with IP10-scFv/EGFRvIII peptide-pulsed, DC-induced CTLs or IP10-scFv compared to those in the control group (*P* < 0.01). However, there were no significant difference in the number of tumor vessels in mice treated with IP10-scFv/EGFRvIII peptide-pulsed, DC-induced CTLs and IP10-scFv (*P* > 0.05). In addition, a large number of CD8^+^ T cells was identified in the intracerebral tumors of mice vaccinated with IP10-scFv/EGFRvIII peptide-pulsed, DC-induced CTLs (*P* < 0.001). In contrast, there was no significant increase in the number of CD8^+^ T cells in the intracerebral tumors from mice treated with IP10-scFv or PBS (*P* > 0.05). These results verified the limited number of CTLs in vivo, especially activated CD8^+^ T cells under normal circumstances.

## Discussion

Adoptive immunotherapy using tumor-reactive T lymphocytes has emerged as a powerful approach for the treatment of bulky, refractory cancer [34, 35]. DC-based vaccination to activate T cells, which is one main form of adoptive immunotherapy, has been shown to effectively mediate antitumor immunity [36]. DC-based immunotherapy can be approached in one of two ways: direct immunization with antigen (tumor-associated proteins, peptides, or tumor lysates) pulsed DCs or adoptive transfer of in vitro expanded CTLs following stimulations with antigen-pulsed DCs. A broad range of immunological defects have been documented in glioma patients, including decreased T cell numbers, impaired T cell responsiveness, and defective signaling after T cell receptor TCR/CD3 stimulation [37]. However, tumor-specific CD8-positive CTLs constitute the most important effector cells for antitumor responses [38, 39]. It has been previously shown that in vitro specific CTLs can be generated using peptide-pulsed autologous DC [40, 41]. However, the interactions between DCs and T lymphocytes are very complicated and may involve different mechanisms [42, 43].

In general, the induction of T lymphocyte activation, proliferation, and function not only requires recognition between the T cell receptor (TCR) and specific major histocomptability complex (MHC) peptide complexes expressed on the surface of APCs (such as DCs and tumor cells), but also the interaction between co-stimulatory molecules on T cells and APCs [44]. Nevertheless, the recognition between the TCR of T lymphocytes and antigen presented by DCs is limited by MHC molecules, and therefore, the killing of target cells by activated CTLs is also limited by MHC molecules. Wu et al. [30] first reported that human DCs pulsed with a 9-mer peptide containing the amino acid fusion site of EGFRvIII protein effectively induced autologous EGFRvIII peptide-specific CTLs in vitro, and that these CTLs effectively killed EGFRvIII-expressing human glioma cells. This 9-mer EGFRvIII peptide is also presented on HLA-A2 molecules, and therefore, the killing of EGFRvIII-expressing glioma cells by peptide-specific CTLs is HLA-A2-restricted. Our findings confirmed the previous work of Wu et al. by finding that the 9-mer EGFRvIII-specific peptide on antigen-pulsed DCs can indeed induces tumor-specific CTLs in vitro.

To date, several strategies have been assessed for creating DC-based cancer vaccines, including: (1) loading DCs with defined TAAs, which is the most commonly applicable strategy, for DC-based vaccination, but has a number of drawbacks [45]; (2) pulsing DCs with tumor DNA/RNA; and (3) generating fusions of DCs and tumor cells. DC/glioma-specific peptides have been confirmed to be a useful strategy to elicit CTL responses and have been shown to be more effective than other types of DC-based vaccines. Therefore, EGFRvIII peptide-pulsed DCs have distinct advantages over other types of DCs, which provide a robust platform for the generation of large numbers of functional antigen-specific CD8+ T cells [46]. We found that these CTLs effectively killed EGFRvIII-expressing human glioma cells. Therefore, future investigations will focus on the potential use of EGFRvIII peptide in DC-based immunotherapy in clinical research.

Nevertheless, efficient immunotherapy requires that cytotoxic T cells not only persist in vivo, but also migrate to and function optimally at the tumor site. The antitumor activity of EGFRvIII peptide-pulsed, DC-induced CTLs is limited in vivo because of tumor immunologic escape [47] and the immunogenicity of tumor deficiency [48]. Although we injected tumor-specific CTLs into the center of the tumor, it is difficult for the cells to infiltrate throughout the tumor from the injection site because of the tumor microenvironment [49], which can inhibit the antitumor activity of CTLs and surround the tumor.

Notably, IP-10 has been shown to be a potent chemotactic factor of CTL [50], and a scFv is an attractive carrier of bioactive molecules into the tumor tissues due to its antigen specificity and small size [51]. Interestingly, IP10 is required for CNS glioma homing as well as the induction of effector CTLs because its receptor, CXCR3, is highly expressed on active T cells. In this study, we tested whether local treatment with IP-10 could effectively EGFRvIII peptide-pulsed, DC-induced CTLs into the tumor and inhibit the growth of glioma in mice. We first constructed the fusion gene IP10-scFv that specifically recognized glioma-specific EGFRvIII antigen, which is commonly expressed on the membrane surface of glioma cells, and effectively recruited glioma-specific CTLs into the tumor to inhibit tumor growth in mice [27]. Following purification and refolding, we found that the recombinant fusion protein IP10-scFv had similar affinity to that of scFv and retained the immunoreactivity of scFv and the chemotactic activity of IP-10. Our results indicate that large numbers of CD8+ T cells infiltrate tumors after antigen-specific T cell transfer following stimulation with EGFRvIII peptide-pulsed DCs. Although this approach was effective in an animal model, the delivery method limits its use in the clinic. Therefore, in future studies we will improve the method of delivery for humans.

## Conclusion

In summary, our data indicated that treatment with IP10-scFv or EGFRvIII peptide-pulsed, DC-induced CTLs significantly enhanced cell infiltration in the brain and expanded the effector T cells in a specific immune response, which was accompanied by enhanced CD8^+^ T cell-mediated cytotoxicity in the brain. In addition, treatment with both IP10-scFv and EGFRvIII peptide-pulsed, DC-induced CTLs synergistically enhanced the therapeutic effect of inhibiting the growth of glioma and prolonged the survival of tumor-bearing mice. Therefore, our findings may provide new insight in glioma-specific immunity and aid in the design of new immunotherapies for treating glioma.

## References

[1] Wick W, Platten M, Meisner C, Felsberg J, Tabatabai G, Simon M (2012). Temozolomide chemotherapy alone versus radiotherapy alone for malignant astrocytoma in the elderly: the NOA-08 randomised, phase 3 trial. Lancet Oncol.

[2] Donson AM, Birks DK, Schittone SA, Kleinschmidt-DeMasters BK, Sun DY, Hemenway MF (2012). Increased immune gene expression and immune cell infiltration in high-grade astrocytoma distinguish long-term from short-term survivors. J Immunol.

[3] Choi D, Kim TG, Sung YC (2012). The past, present, and future of adoptive T cell therapy. Immune Netw.

[4] Ishida A, Tanaka H, Hiura T, Miura S, Watanabe S, Matsuyama K (2007). Generation of anti-tumour effector T cells from naive T cells by stimulation with dendritic/tumour fusion cells. Scand J Immunol.

[5] Vesely MD, Kershaw MH, Schreiber RD, Smyth MJ (2011). Natural innate and adaptive immunity to cancer. Annu Rev Immunol.

[6] Carrasco J, Van Pel A, Neyns B, Lethe B, Brasseur F, Renkvist N (2008). Vaccination of a melanoma patient with mature dendritic cells pulsed with MAGE-3 peptides triggers the activity of nonvaccine anti-tumor cells. J Immunol.

[7] Bigner SH, Humphrey PA, Wong AJ, Vogelstein B, Mark J, Friedman HS (1990). Characterization of the epidermal growth factor receptor in human glioma cell lines and xenografts. Cancer Res.

[8] Kuan CT, Wikstrand CJ, Bigner DD (2001). EGF mutant receptor vIII as a molecular target in cancer therapy. Endocr Relat Cancer.

[9] Emrich JG, Brady LW, Quang TS, Class R, Miyamoto C, Black P (2002). Radioiodinated (I-125) monoclonal antibody 425 in the treatment of high grade glioma patients: ten-year synopsis of a novel treatment. Am J Clin Oncol.

[10] Peng W, Zhao G, Ma Y, Yu H, Wang X (2011). Dendritic cells transfected with PEG10 recombinant adenovirus elicit anti-tumor immune response in vitro and in vivo. Vaccine.

[11] Noh YW, Jang YS, Ahn KJ, Lim YT, Chung BH (2011). Simultaneous in vivo tracking of dendritic cells and priming of an antigen-specific immune response. Biomaterials.

[12] Wierecky J, Mueller M, Brossart P (2006). Dendritic cell-based cancer immunotherapy targeting MUC-1. Cancer Immunol Immunother.

[13] Ilias Basha H, Tiriveedhi V, Fleming TP, Gillanders WE, Mohanakumar T (2011). Identification of immunodominant HLA-B7-restricted CD8+ cytotoxic T cell epitopes derived from mammaglobin-A expressed on human breast cancers. Breast Cancer Res Treat.

[14] Wintermeyer P, Gehring S, Eken A, Wands JR (2010). Generation of cellular immune responses to HCV NS5 protein through in vivo activation of dendritic cells. J Viral Hepat.

[15] Kono M, Nakamura Y, Suda T, Uchijima M, Tsujimura K, Nagata T (2012). Enhancement of protective immunity against intracellular bacteria using type-1 polarized dendritic cell (DC) vaccine. Vaccine.

[16] Yoon SH, Yun SO, Park JY, Won HY, Kim EK, Sohn HJ (2009). Selective addition of CXCR3(+) CCR4(−) CD4(+) Th1 cells enhances generation of cytotoxic T cells by dendritic cells in vitro. Exp Mol Med.

[17] Heimberger AB, Archer GE, Crotty LE, McLendon RE, Friedman AH, Friedman HS (2002). Dendritic cells pulsed with a tumor-specific peptide induce long-lasting immunity and are effective against murine intracerebral melanoma. Neurosurgery.

[18] Rossi D, Zlotnik A (2000). The biology of chemokines and their receptors. Annu Rev Immunol.

[19] Sun H, Kundu N, Dorsey R, Jackson MJ, Fulton AM (2001). Expression of the chemokines IP-10 and Mig in IL-10 transduced tumors. J Immunother.

[20] Fujita M, Zhu X, Ueda R, Sasaki K, Kohanbash G, Kastenhuber ER (2009). Effective immunotherapy against murine gliomas using type 1 polarizing dendritic cells—significant roles of CXCL10. Cancer Res.

[21] Enderlin M, Kleinmann EV, Struyf S, Buracchi C, Vecchi A, Kinscherf R (2009). TNF-alpha and the IFN-gamma-inducible protein 10 (IP-10/CXCL-10) delivered by parvoviral vectors act in synergy to induce antitumor effects in mouse glioblastoma. Cancer Gene Ther.

[22] Kioi M, Seetharam S, Puri RK (2008). Targeting IL-13Ralpha2-positive cancer with a novel recombinant immunotoxin composed of a single-chain antibody and mutated Pseudomonas exotoxin. Mol Cancer Ther.

[23] Modjtahedi H, Moscatello DK, Box G, Green M, Shotton C, Lamb DJ (2003). Targeting of cells expressing wild-type EGFR and type-III mutant EGFR (EGFRvIII) by anti-EGFR MAb ICR62: a two-pronged attack for tumour therapy. Int J Cancer.

[24] Li L, Quang TS, Gracely EJ, Kim JH, Emrich JG, Yaeger TE (2010). A phase II study of anti-epidermal growth factor receptor radioimmunotherapy in the treatment of glioblastoma multiforme. J Neurosurg.

[25] Lorimer IA, Keppler-Hafkemeyer A, Beers RA, Pegram CN, Bigner DD, Pastan I (1996). Recombinant immunotoxins specific for a mutant epidermal growth factor receptor: targeting with a single chain antibody variable domain isolated by phage display. Proc Natl Acad Sci U S A.

[26] Wang X, Lu XL, Zhao HY, Zhang FC, Jiang XB (2013). A novel recombinant protein of IP10-EGFRvIIIscFv and CD8(+) cytotoxic T lymphocytes synergistically inhibits the growth of implanted glioma in mice. Cancer Immunol Immunother.

[27] Kuan CT, Reist CJ, Foulon CF, Lorimer IA, Archer G, Pegram CN (1999). 125I-labeled anti-epidermal growth factor receptor-vIII single-chain Fv exhibits specific and high-level targeting of glioma xenografts. Clin Cancer Res.

[28] Schmittling RJ, Archer GE, Mitchell DA, Heimberger A, Pegram C, Herndon JE (2008). Detection of humoral response in patients with glioblastoma receiving EGFRvIII-KLH vaccines. J Immunol Methods.

[29] Mirecka EA, Rudolph R, Hey T (2006). Expression and purification of His-tagged HPV16 E7 protein active in pRb binding. Protein Expr Purif.

[30] Wu AH, Xiao J, Anker L, Hall WA, Gregerson DS, Cavenee WK (2006). Identification of EGFRvIII-derived CTL epitopes restricted by HLA A0201 for dendritic cell based immunotherapy of gliomas. J Neurooncol.

[31] Calzascia T, Masson F, Di Berardino-Besson W, Contassot E, Wilmotte R, Aurrand-Lions M (2005). Homing phenotypes of tumor-specific CD8 T cells are predetermined at the tumor site by crosspresenting APCs. Immunity.

[32] Michael NP, Chester KA, Melton RG, Robson L, Nicholas W, Boden JA (1996). In vitro and in vivo characterisation of a recombinant carboxypeptidase G2::anti-CEA scFv fusion protein. Immunotechnology.

[33] Xi Y, Yuan Z, Zhang H, Guan H, Kong F, Liu N (2006). Molecular construction and characterization of a novel exotoxin fusion protein that selectively blocks the B7:CD28 costimulatory signal system. J Immunother.

[34] Gilboa E. DC-based cancer vaccines. J Clin Invest. 2007;117(5):1195–203. doi:10.1172/JCI31205. 10.1172/JCI31205 PMC1857263 17476349

[35] Gattinoni L, Powell DJ, Rosenberg SA, Restifo NP (2006). Adoptive immunotherapy for cancer: building on success. Nat Rev Immunol.

[36] Siders WM, Garron C, Shields J, Kaplan JM (2009). Induction of antitumor immunity by semi-allogeneic and fully allogeneic electrofusion products of tumor cells and dendritic cells. Clin Transl Sci.

[37] Waldron JS, Yang I, Han S, Tihan T, Sughrue ME, Mills SA (2010). Implications for immunotherapy of tumor-mediated T-cell apoptosis associated with loss of the tumor suppressor PTEN in glioblastoma. J Clin Neurosci.

[38] Rammensee HG, Friede T, Stevanoviic S (1995). MHC ligands and peptide motifs: first listing. Immunogenetics.

[39] Ranganathan S, Tong JC (2007). A practical guide to structure-based prediction of MHC-binding peptides. Methods Mol Biol.

[40] Paczesny S, Banchereau J, Wittkowski KM, Saracino G, Fay J, Palucka AK (2004). Expansion of melanoma-specific cytolytic CD8+ T cell precursors in patients with metastatic melanoma vaccinated with CD34+ progenitor-derived dendritic cells. J Exp Med.

[41] Perroud MW, Honma HN, Barbeiro AS, Gilli SC, Almeida MT, Vassallo J (2011). Mature autologous dendritic cell vaccines in advanced non-small cell lung cancer: a phase I pilot study. J Exp Clin Cancer Res.

[42] Chen JH, Yu YS, Chen XH, Liu HH, Zang GQ, Tang ZH (2012). Enhancement of CTLs induced by DCs loaded with ubiquitinated hepatitis B virus core antigen. World J Gastroenterol.

[43] Li A, Xiong S, Lin Y, Liu R, Chu Y (2011). A high-affinity T-helper epitope enhances peptide-pulsed dendritic cell-based vaccine. DNA Cell Biol.

[44] Tiriveedhi V, Sarma NJ, Subramanian V, Fleming TP, Gillanders WE, Mohanakumar T (2012). Identification of HLA-A24-restricted CD8(+) cytotoxic T-cell epitopes derived from mammaglobin-A, a human breast cancer-associated antigen. Hum Immunol.

[45] Koido S, Homma S, Hara E, Namiki Y, Ohkusa T, Gong J (2010). Antigen-specific polyclonal cytotoxic T lymphocytes induced by fusions of dendritic cells and tumor cells. J Biomed Biotechnol.

[46] Zhi-Iong Ma J, Yang J, Qin JS, Richter A, Perret R, El-Deiry WS (2012). Inefficient boosting of antitumor CD8(+) T cells by dendritic-cell vaccines is rescued by restricting T-cell cytotoxic functions. Oncoimmunology.

[47] Ichiki Y, Hanagiri T, Takenoyama M, Baba T, Nagata Y, Mizukami M (2012). Differences in sensitivity to tumor-specific CTLs between primary and metastatic esophageal cancer cell lines derived from the same patient. Surg Today.

[48] Cho SJ, Kim JS, Kim JM, Lee JY, Jung HC, Song IS (2008). Simvastatin induces apoptosis in human colon cancer cells and in tumor xenografts, and attenuates colitis-associated colon cancer in mice. Int J Cancer.

[49] Weigelin B, Krause M, Friedl P (2011). Cytotoxic T lymphocyte migration and effector function in the tumor microenvironment. Immunol Lett.

[50] Ramanathan S, Gagnon J, Dubois S, Forand-Boulerice M, Richter MV, Ilangumaran S (2009). Cytokine synergy in antigen-independent activation and priming of naive CD8+ T lymphocytes. Crit Rev Immunol.

[51] Liu M, Guo S, Stiles JK (2011). The emerging role of CXCL10 in cancer (review). Oncol Lett.

